# Sensory Enrichment and Deprivation During Development: Limited Effects on the Volumes of CNS Neuropils in Two Spiders With Different Ecology

**DOI:** 10.1002/cne.70102

**Published:** 2025-11-20

**Authors:** Philip O. M. Steinhoff, Pierick Mouginot, Gabriele B. Uhl

**Affiliations:** ^1^ Zoological Institute and Museum, General and Systematic Zoology University of Greifswald Greifswald Germany; ^2^ Behavioral Ecology and Fish Population Biology, INRAE UPPA UMR 1224 ECOBIOP Saint‐Pée‐sur‐Nivelle France

**Keywords:** brain plasticity, cursorial, family effect, neuropil, neuroplasticity, RRID:SCR_011649, spiders, web‐building

## Abstract

Neuroplasticity is a core property of animal nervous systems, enabling structural changes in the brain in response to environmental stimuli or internal processes such as learning. Among spiders—a diverse group of predators—neuroanatomy varies with hunting strategy: stationary species that build capture webs differ from cursorial species that hunt without webs, reflecting reliance on distinct sensory modalities. While neuroplasticity has been documented in cursorial jumping spiders, its direct drivers remain unclear. In this study, we tested how sensory input influences the central nervous system (CNS) and whether stationary and cursorial hunters differ in their plastic responses. Using sensory deprivation and enrichment, we reared spiders under four treatments: control (CON), vibratory enrichment (VIB), visual enrichment (VIS), and combined enrichment (VISVIB). We examined the stationary hunter *Parasteatoda tepidariorum* and the cursorial hunter *Marpissa muscosa*. We predicted that enrichment would enlarge neuropil volumes in modality‐specific brain regions, with stronger vibratory effects in *P. tepidariorum* and stronger visual effects in *M. muscosa*. Contrary to our expectations, sensory enrichment did not increase the volume of the corresponding CNS neuropils in either species. Although certain neuropils showed significant differences in specific groups, no clear causal link to sensory input emerged. Instead, a substantial proportion of the variation in neuropil volume was explained by family effects (shared maternal origin). We discuss these findings in the context of potential mechanisms underlying environmental plasticity in the spider brain.

## Graphical abstract

Sensory enrichment did not increase modality‐specific brain region volumes in either *Parasteatoda tepidariorum* or *Marpissa muscosa*; variation was largely explained by shared maternal origin, suggesting that genetic or developmental factors may outweigh environmental sensory input in shaping spider brain structure.

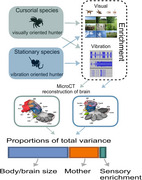

## 1 Introduction

Changing environmental conditions may have selected for adaptive flexibility of the brain in animals, and, indeed, neuroplasticity is a ubiquitous feature of all nervous systems. Neuroplasticity can occur both during an individual's development (i.e., experience‐expectant plasticity) and during the adult phase (i.e., experience‐dependent plasticity; Kolb and Gibb 2014). Neuroplasticity as a response to learning and memory formation has been explored most intensively in vertebrates (Burns et al. 2009; Gogolla et al. 2007; Guay and Iwaniuk 2008; Joyce and Brown 2022; Kihslinger 2006; Orije and Van der Linden 2022; Rosenzweig and Bennett 1996) but has also been observed in adult individuals of different insect species (Anton and Rössler 2020 review on olfactory circuit plasticity; Fahrbach et al. 2003, 1998; Fahrbach and Van Nest 2016; Friedel and Barth 1997; Withers et al. 2008, 1993; Yilmaz et al. 2016). Changes in the structure of the brain under different environmental conditions during development have been identified in insects and spiders (Groh et al. 2006, 2004; Montgomery et al. 2016; Steinhoff et al. 2018). However, the mechanisms underlying the structural changes in the nervous system remain poorly understood, as it is challenging to isolate the effect of the sensory input itself from cognitive processes, learning, spatial navigation, or other types of sensory input (Eriksson et al. 2019).

Spiders are a large group of mesopredators with diverse sensory ecologies (Foelix 2011) that have led to adaptive changes in the degree of investment in sensory systems to optimize behavioral performance under different contexts and according to lifestyle. While many spiders have evolved capture webs for foraging and rely heavily on vibratory cues, other species are cursorial and predominantly utilize visual cues to capture prey (Barth 1985; Foelix 2011). Neuroplasticity has not yet been studied in much detail in spiders, although spiders are an excellent species group for investigating sensory detection, neural and cognitive processes (Jackson and Cross 2011; Liedtke and Schneider 2014; Peckmezian and Taylor 2017; Steinhoff et al. 2018; Nelson 2025). They are diverse in their foraging ecology, encompassing cursorial hunters and sit‐and‐wait predators. Environmental conditions and specific lifestyles likely shape the sensory systems and degree of brain plasticity exhibited by a species. This phenomenon is exemplified by males and females of a net‐casting spider *Deinopis spinosa*, in which juveniles of both sexes utilize the same foraging mode, but after the final molt to maturity, males become cursorial, search for a female, and cease foraging. Correspondingly, brain structures in adult males differ significantly from those of adult females (Stafstrom et al. 2017), demonstrating remarkable neural plasticity that occurs from one molt to the next. That spider brain development can be very plastic depending on environmental conditions was demonstrated in a study on a salticid spider (Steinhoff et al. 2018). In this study, the environmental conditions under which spiders were reared were (1) deprived, (2) physically enriched, and (3) socially enriched (multiple spiders together in a larger enclosure). Spiders from the deprived group had smaller higher‐order neuropils and total brain volumes than spiders from the enriched group (Steinhoff et al. 2018), providing evidence that environmental stimuli can influence brain development. While these studies demonstrate a significant potential for neural plasticity during development, knowledge is lacking on the extent to which spider brains respond to input from various sensory modalities and whether these plastic responses differ between species with diverse sensory ecologies. Specifically, the brains of cursorial spiders that rely on vision (active cursorial hunters) and those that primarily rely on vibration (sit‐and‐wait predators in their web) are adapted to their primary mode of sensory input and are thus expected to exhibit neuronal plasticity in response to stimulation and deprivation.

A notable difference between spiders with different lifestyles is the size of the eyes (Morehouse 2020). Eye size has been shown to correlate with differences in the visual pathways of the brain (Hanström 1921; Long 2021; Steinhoff et al. 2024, 2020). The majority of spiders possess four pairs of eyes, of which one pair (the principal eyes or anterior median eyes, AMEs) is structurally and functionally distinct from the other three pairs (secondary eyes: posterior median eyes, PME, anterior lateral eyes, ALEs, and posterior lateral eyes, PLEs; Morehouse 2020). While the AMEs have a movable retina and are used for object recognition, the secondary eyes have a fixed retina and are used for movement detection (Morehouse 2020). The central nervous system (CNS) of spiders is a highly fused mass of nervous tissue situated in the prosoma and consists of an anterior brain and a posterior ventral nerve cord (VNC; Steinhoff et al. 2017). The brain contains the first‐ and second‐order visual neuropils of the AME and a variable number of visual neuropils that serve the secondary eyes (Steinhoff et al. 2020; Strausfeld and Barth 1993). These neuropils process primary sensory information from the eyes (Steinhoff et al. 2020) and are connected to the arcuate body (AB), a crescent‐shaped higher‐order neuropil at the posterior rim of the brain (Strausfeld et al. 1993; Steinhoff et al. 2020a). The visual neuropils of the secondary eyes are also connected to the mushroom bodies (MBs) in some species, a paired higher‐order neuropil of the brain (Steinhoff et al. 2020; Strausfeld and Barth 1993). The central part of the brain is composed predominantly of the protocerebral neuropil (ProtoN) and tracts, connecting it to the VNC (Babu and Barth 1984; Steinhoff et al. 2024). The posterior part of the brain is formed by the cheliceral and the pedipalpal neuropils (PdNs), while the VNC comprises the opisthosomal neuropil (OpN), four paired leg neuropils (LegN), and a central part consisting of tracts and neuropilar regions (Babu and Barth 1984; Steinhoff et al. 2024). The LegNs receive and process mechanosensory (i.e., vibrational) and also chemosensory information (Babu and Barth 1989).

In this study, we investigated whether environmental stimuli during early postembryonic development influence the CNS structure in spiders and whether these effects differ by hunting strategy. We used the cursorial hunter, *Marpissa muscosa*, and the stationary web builder, *Parasteatoda tepidariorum*, because these two species differ strongly in their hunting strategies, they can be reared and kept in the laboratory, and published data on the anatomy of their CNSs exist (Steinhoff et al. 2024). We reared the spiders under four conditions: visual enrichment (VIS), vibratory enrichment (VIB), combined enrichment (VISVIB), and a deprived control (CON) with minimal sensory input. We examined which CNS neuropils were affected by these environments and analyzed other sources of variation, such as body and brain size and family effects. We hypothesized that species‐specific sensory ecologies would shape neuroplastic responses, with (i) larger visual neuropils (AM1 and SecVN1) in VIS and VISVIB treatments in *M. muscosa* and (ii) larger LegN in VIB and VISVIB treatments in both species, with stronger effects in *P. tepidariorum*. Beyond exploring neuroplasticity in relation to the environment and lifestyle, this study also aimed to shed light on the functional roles of specific spider brain regions.

## 2 Material and Methods

### 2.1 Experimental Animals and Housing

Adult females of *M. muscosa* (Clerck, 1757) were collected in and near Greifswald (Germany). They were kept in plastic boxes (145 × 110 × 68 mm), supplemented with paper tissue. Approximately one week after collection, the females produced egg sacs. Spiderlings hatched within three weeks from the egg sac and were transferred to individual plastic boxes (145 × 110 × 68 mm). The bottom and long sides of the plastic boxes were painted with brown paint. A window was cut into one of the short sides and covered with gauze. This side is termed “front side.” The opposite short side (“back side”) was left intact so that the spiders could see through the transparent plastic (see below). Each spider received an ID and was randomly assigned to one of four treatment groups: visual treatment (VIS), vibratory treatment (VIB), visual and vibratory treatment (VISVIB), and control treatment (CON).

Adult specimens of *P. tepidariorum* were collected from the greenhouses of the botanical garden at the University Greifswald and reared in climate chambers (26°C, 80% humidity, 12/12 h light cycle). Individual spiders were housed in plastic boxes (145 × 110 × 68 mm) supplemented with paper tissue. In cases where females did not produce an egg sac within one week, they were paired overnight with a male. Upon emergence of spiderlings from an egg sac, they were transferred to individual boxes and randomly assigned to one of the four treatments (see above). Spiders were provided with a regular amount of *Drosophila* flies, which increased with developmental stage from 3 to 15 per week.

### 2.2 General Experimental Procedure

The rearing boxes of *M. muscosa* were placed on wooden shelves. We built eight shelves that each housed 30 rearing boxes (five rows with six boxes each). Initially, 60 spiderlings from 14 mothers were assigned to each treatment group (VIS, VIB, VISVIB, CON). The shelves were designed to precisely accommodate the monitor that played the visual stimuli (see below) within the frame, directly in front of the short back side (without gaze) of the boxes. For *P. tepidariorum*, 30 spiderlings from 11 mothers were assigned to each treatment group. The boxes of *P. tepidariorum* were oriented vertically, so that spiders could build their capture webs. Rearing boxes with spiders from the same treatment were positioned next to each other, separated by cardboard spacers and secured with straps.

Spiders received cues of prey (videos and vibrations) once a week on the day they were fed. The other visual and vibratory stimuli (see below) were presented in random order, with spiders receiving visual and vibratory playback on 4 days of the week for a minimum of 4 and a maximum of 14 h, depending on the season. Playback started after sunrise and ended before sunset. For *M. muscosa*, the experiments ran from beginning of July 2019 to mid‐May 2020 (10.5 months) when all spiders had reached the subadult stage, while in *P. tepidariorum* the experiment lasted from 1st of March 2020 to the end of May 2020 (3 months) when all spiders had reached the adult stage. The difference in duration of the experiments reflects the different maturation time and life expectancy of the two species. After termination of the experiments, spiders were chemically fixed for the analysis of the neuropil volumes (see below).

### 2.3 Video Playback

Video playback is a well‐established method for behavioral research in cursorial hunting spiders (Clark and Uetz 1990; Menda et al. 2014; Peckmezian and Taylor 2015; Uetz et al. 2017). Habituation has been shown to affect behavior and synaptic activity of the brain (Carew et al. 1972; Engel and Wu 2009; Humphrey et al. 2018; Melrose et al. 2019). Therefore, we created diverse visual stimuli and presented them to the spiders in a semirandom order. Visual stimuli were videos of moving prey (drawings of flies prepared using CorelDraw according to Menda et al. (2014); Figure S1a) or videos of moving jumping spiders exhibiting various body postures (drawings prepared using CorelDraw according to Menda et al. 2014; Figure S1a). Videos were created as GIF‐files by stitching together single images that served as individual frames, using GIMP 2.1.0. The package magick in R 3.5.3 was used to combine individual videos into a single mosaic video with 6 × 5 mosaic tiles (one tile per spider), with each tile showing identical video content (Figure S1c). Further visual stimuli consisted of still images of aspects of the environment (soil, grass, sky, etc., Figure S1b) that were shown consecutively to the spiders in 10 s or 1 min intervals. All visual stimuli were presented in loop mode on 27 in. monitors (Acer) in full‐screen mode, ensuring that each tile possessed dimensions precisely matching the transparent back of the rearing boxes. A VLC media player on Raspberry Pi 3 computers was used to run the videos. Preliminary tests with *M. muscosa* spiders collected from natural habitats showed that they reacted to the video playback of moving flies with characteristic behaviors that precede a prey attack (i.e., stalking).

### 2.4 Vibratory Playback

The setup for the vibratory playback closely follows the methods described and used by Uetz et al. (2017). Spiders in the group VIB and VISVIB received vibratory input via piezoceramic elements (KEPO FT‐35T‐2.9AL‐888; voltage: 30 V; 35 mm diameter) that were attached to the shelf boards, contacting the bottom of the rearing boxes. Vibratory input consisted of recordings of different prey items (*Drosophila* sp., *Lucilia* sp., and *Calliphora* sp. walking and buzzing) and environmental noise. Vibrations were recorded using a laser vibrometer (PDV‐100, PolytecGmbH), and played back using an amplifier (Pyle Mini 2 × 40 W Stereo Power Amplifier) that was connected to the piezoceramic elements with speaker wire. VLC media player on Raspberry Pi 3 computers was used to play the vibrations in loop mode. The output was split into all 30 boxes in one shelf using luster terminals. Played‐back vibrations were validated by recording them from the spider rearing box with the laser vibrometer and comparing the waveforms (amplitude and pattern) to the original recordings using Audacity 2.1.1 (Figure S2).

### 2.5 Sample Preparation and MicroCT Analysis

Prosomata were fixed overnight at 4°C in 2% PFA. Samples were moved into a mixture of 80% methanol and 20% DMSO on a shaker for 12 h and then stored at −16°C in 99.8% methanol. Before scanning, samples were transferred to a 2% iodine solution (iodine, resublimated [Carl Roth, X864.1] in 99.8% methanol) for 24 h to enhance tissue contrast. Samples were washed three times in 99.8% methanol, mounted in fresh 99.8% methanol, and scanned. Wet scans were used to avoid tissue shrinkage (Rivera Quiroz and Miller 2022) and methanol to increase contrast compared to ethanol. MicroCT scans were carried out with an optical laboratory‐scale X‐ray microscope (Zeiss XradiaXCT‐200, RRID: SCR_011649; Sombke et al. 2015). Scans were performed with a 4 × or 10 × objective lens unit using the following settings: 40 kV, 8 W, 200 µA and exposure times between 1 and 3s. Scans took between 1 and 2 h and resulted in pixel sizes between 2.13 µm and 2.4 µm. Reconstruction of tomographic projections was done using the XMReconstructor software (Zeiss), resulting in image stacks (TIFF format). All scans were performed using Binning 2 for noise reduction (summarizing 4 pixels) and were reconstructed with full resolution (using Binning 1; see Figure S3 for an example of the grayscale TIFF‐images generated by the microCT).

### 2.6 Volume Calculation and Statistical Analysis

**FIGURE 1:**
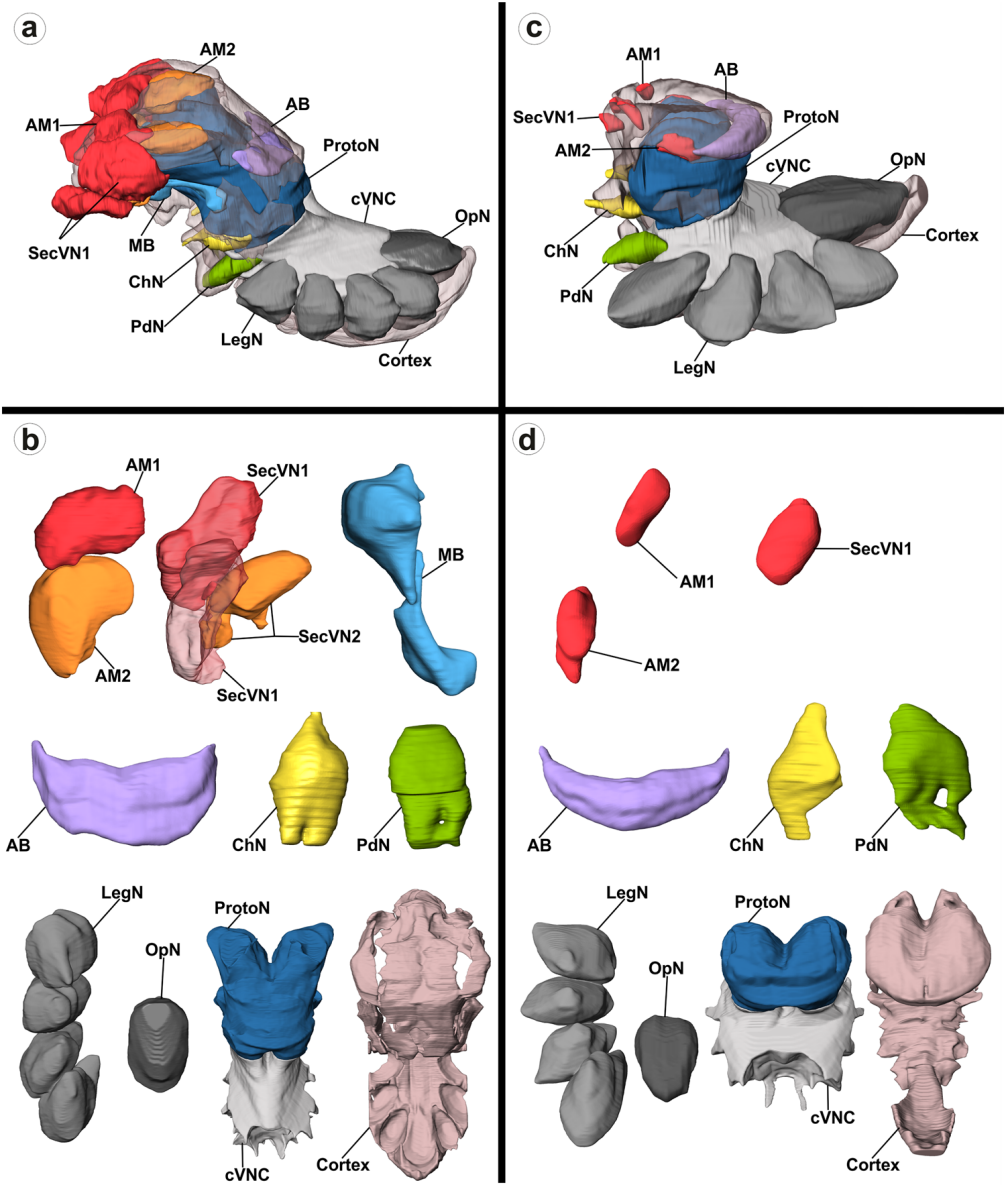
General characteristics of the cortex and all CNS neuropils of *M. muscosa* (A,B) and *P. tepidariorum* (C,D), reconstructed from MicroCT data. (A,C) Overview of the CNS with all reconstructed structures highlighted in different colors. Cortex transparent for clarity. (B,D) All individual neuropils in dorsal view, only the left neuropil is shown for bilaterally paired neuropils. AB, arcuate body; AM1, first‐order visual neuropil of the anterior median eyes; AM2, second‐order visual neuropil of the anterior median eyes; ChN, cheliceral neuropil; CNS, central nervous system (sum of all reconstructed neuropils); Cortex, perikaryal rind; cVNC central part of the ventral nerve cord; LegN, leg neuropils; MB, mushroom bodies; OpN, opisthosomal neuropil; PdN, pedipalpal neuropil; ProtoN, protocerebral neuropil; ProtoN_cVNC, protocerebral and central ventral nerve cord neuropils combined; SecVN1, first‐order visual neuropils of all secondary eyes; SecVN2, second‐order visual neuropils of all secondary eyes.

The prosoma, cortex, and all discernible neuropils in the CNS were reconstructed, and their volumes were calculated using AMIRA 5.4.5 and 6.0.0 (Visualization Science Group, FEI; Figure 1). All volume reconstructions were carried out by the same observer, tracings were done by hand, and no automatic segmentation was used. Forty‐five individuals were reconstructed for each species. Specimens were equally distributed among the treatment groups, with 11 individuals per treatment, except for the vibration stimulus group in *Marpissa* and the Visual stimulus group in *Parasteatoda*, with 12 individuals each. All statistical analyses were performed in R 4.4.1.

To assess the effect of vibration, vision, or both stimuli on each neuropil volume, we built a mixed model including the stimulus treatment as a fixed explanatory variable and “mother ID” as a random effect to account for among‐family similarity. We fitted a mixed effect model for each volume of interest; however, to avoid pseudoreplication by of analyzing different neuropils of the same individual, we corrected for size. For the neuropil volumes, we included the CNS volume and its quadratic effect as covariates. Thus, effects are assessed for an average CNS volume. For the CNS volume, we included the prosoma size and its quadratic effect as covariates instead of the CNS volume. Thus, treatment effects are assessed for an average prosoma size. To correct for batch effect and ease model convergence, we first fitted a linear model for each neuropil with its volume as the dependent variable and observer as the explanatory variable. We used the residuals of each model as the dependent variable for each volume of interest.

We fitted the mixed models with the lme4 package (Bates et al. 2015) and checked linearity assumptions with the DHARMa package (Hartig 2022). In order to match linear assumptions, we included a polynomial effect of degree 3 instead of 2 for the SecVN2, OpN, and ProtoN VNC models, and removed one outlier for the LegN and OpN models, two outliers for the AB model, and three outliers (one in each treatment) for the ProtoN VNC model. We followed the same procedure for *Parasteatoda*. To meet linear assumptions, we added a polynomial effect of degree 3 for the AM2 model, and we removed one outlier for both the AM1 and AM2 models. Finally, each of the three treatments is compared to the control group, and thus we interpret the results in terms of differences relative to the control group. A negative coefficient means that the treatment group has a smaller neuropil volume compared to the control group, and conversely for a positive coefficient. We normalized (centered and reduced) all continuous variables, which allowed us to compare the effects among neuropil volumes and species.

We further decomposed the total variance of the volume of interest by estimating the components of variance attributable to the random and fixed effects of each mixed model. We partitioned the fixed effect component into the variance contributions of each predictor by estimating the semipartial components with the partR2 package (Stoffel et al. 2021) and the 95% confidence interval with 1000 bootstraps. The variance component due to the random effect corresponds to an among‐family similarity that may originate from a mother effect (genetic and/or maternal effects) or an early environment similarity (cocoon effect). The variance component due to the fixed effects comprises two elements: the variance attributable to the CNS size (or body size), corresponding to the body constraints, and the component due to the treatment, representing the environmental effect on the neuropil volume.

### 2.7 Image Processing and Nomenclature

Drawings and figure plates were produced using CorelDRAW 20.1. Images were processed in Corel Paint Shop Pro using global contrast and brightness adjustment features. The terminology used for CNS structures follows that of Richter et al. (2010). Spider‐specific CNS structures are named according to the terminology used by Steinhoff et al. (2024).

## 3 Results

### 3.1 Neuroanatomy of *M. muscosa* and *P. tepidariorum*

This section provides a general description of the neuropils that were reconstructed and used for volumetric analysis in *M. muscosa* and *P. tepidariorum* (see Steinhoff et al. 2024 for a detailed description of the neuroanatomy of both species). The brain of *M. muscosa* is characterized by large visual neuropils, that are located anteriorly (Figure 1A). The first‐order visual neuropils of the principal eyes (AM1) are thick and slightly crescent‐shaped (Figure 1B). Immediately posterior to the AM1 is the AM2 (second‐order visual neuropil of the principal eyes), which is a large, roundish structure elongated toward the posterior region of the brain (Figure 1A,B). The three secondary pairs of eyes provide three first‐order visual neuropils (SecVN1) that are situated anterior‐laterally and encompass two second‐order visual neuropils (SecVN2), which are positioned more centrally within the brain (Figure 1A,B). Proximal to SecVN2 are the MB, a higher‐order neuropil that consists of two tightly adjoining substructures (Figure 1B). At the posterior rim of the brain and ensheathed by a layer of cell bodies is the AB, another higher‐order neuropil (Figure 1A,B). The rest of the brain is the large protocerebral neuropil (ProtoN), which predominantly consists of tracts and commissures (Figure 1A,B). Ventral to the brain are the cheliceral neuropil (ChN) and the PdN, followed by the four LegN and the OpN (Figure 1A,B). The latter two are situated next to the central ventral nerve cord (cVNC), which consists of tracts, commissures, and neuropilar areas (Figure 1A,B). The CNS is surrounded by a layer of cell bodies of varying thickness (cortex; Figure 1A,B). While Cortex, OpN, LegN, cVNC, PdN, and ChN appear to be highly similar in *P. tepidariorum* (Figure 1C,D), there are several significant differences in the brain compared to *M. muscosa*. *P. tepidariorum* possesses two successive neuropils that serve the principal eyes (AM1, AM2), but only a single small visual neuropil that serves all secondary eyes (SecVN1; Figure 1C,D). We did not detect MB in *P. tepidariorum*, but a prominent AB (Figure 1C,D).

### 3.2 Effect of Sensory Enrichment on the Neuropil Volume in *M. muscosa* and *P. tepidariorum*

**FIGURE 2:**
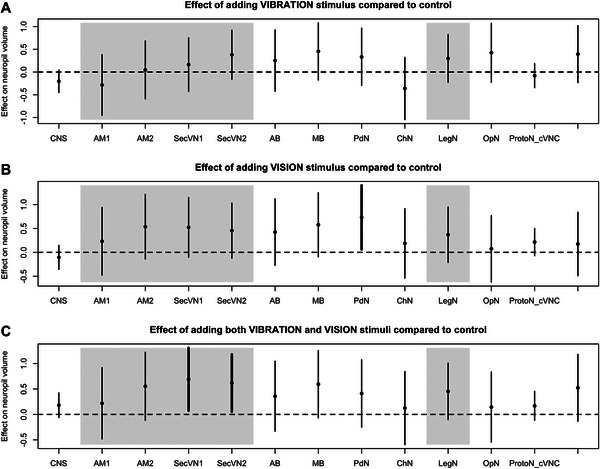
Effect of stimuli treatments on neuropil volumes in *Marpissa muscosa*. The effect of each stimulus treatment (vibratory (A), vision (B), vibratory and vision (C)) compared to the control group is represented for each neuropil volume with its 95% confidence interval. The vision‐related neuropils (AM1, AM2, SecVN1, SecVN2) and the vibration‐related neuropil (LegN) are highlighted by the gray areas. Confidence intervals that do not overlap with zero are interpreted as showing a significant difference with the control group and are visualized with a thicker line. For an explanation of the abbreviations, see Fig. 1.

**FIGURE 3:**
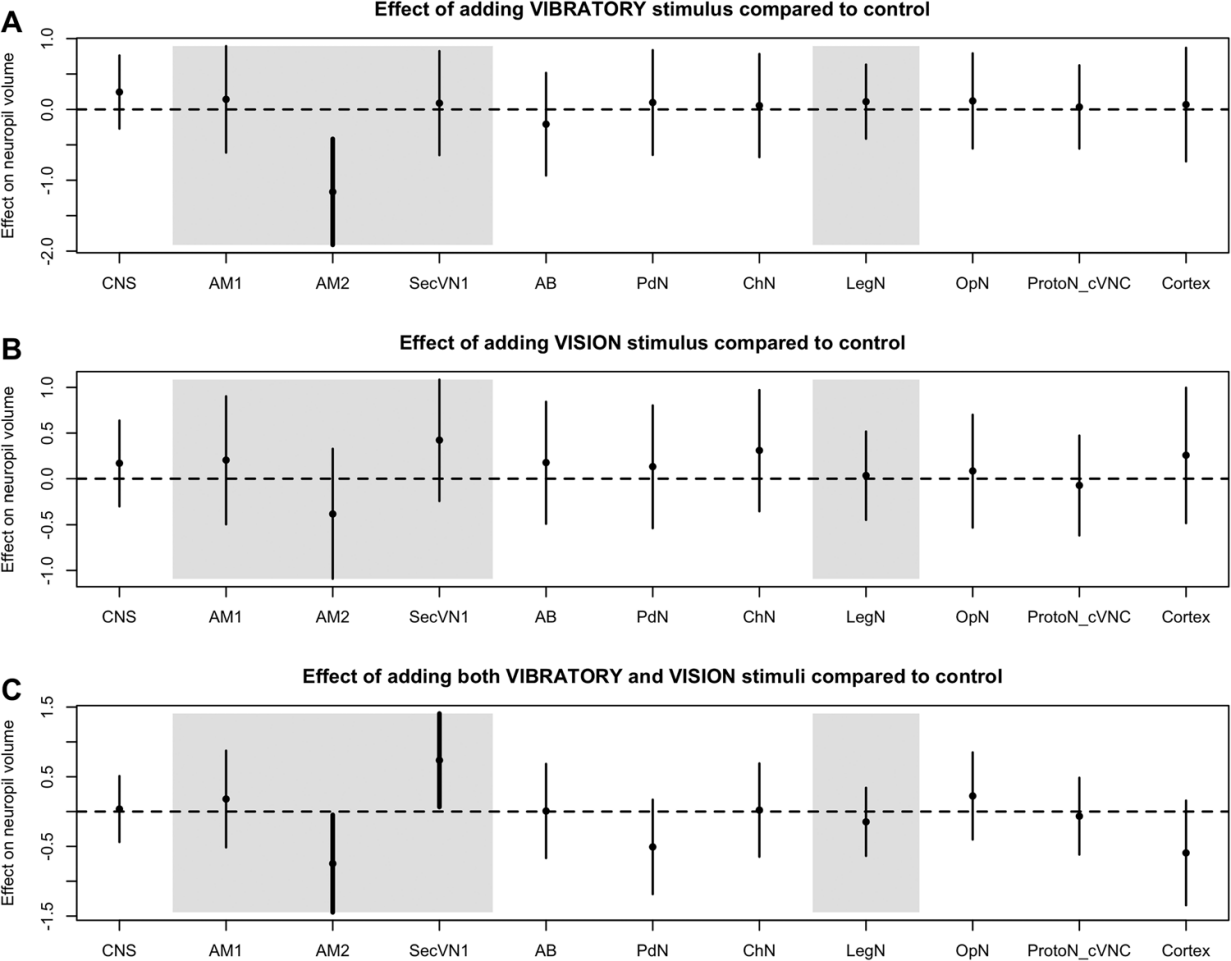
Effect of stimulus treatment on neuropil volumes in *Parasteatoda tepidariorum*. The effect of each stimulus treatment (vibratory (A), vision (B), vibratory, and vision (C)) compared to the control group is represented for each neuropil volume with its 95% confidence interval. The vision‐related neuropils (AM1, AM2, SecVN1) and the vibration‐related neuropil (LegN) are highlighted by the gray areas. Confidence intervals that do not overlap with zero are interpreted as showing a significant difference from the control group and are represented by a thicker line. For an explanation of the abbreviations, see Fig. 1.

We assessed the effect of each stimulus treatment on neuropil volumes by comparing the volumes for each neuropil to those of the control group. In *M. muscosa*, we found three neuropil volumes that significantly differed from the control group (Figure 2 and Table S1). The vision stimulus (VIS) positively affected the volume of the PdN neuropil, which exhibited a larger size compared to the control group. The vibratory and visual stimuli group (VISVIB) showed a positive effect on the SecVN1 and SecVN2 neuropil volumes. In *P. tepidarum*, two visual neuropils were significantly affected in treatments where a vibratory stimulus was present (VIB or VISVIB treatments; Figure 3 and Table S2). The volume of the AM2 neuropil, the second‐order visual neuropil of the principal eyes, was negatively affected by both the VIB and VISVIB treatments. The volume of SecVN1, the first‐order neuropil of the secondary eyes, was positively affected by the VISVIB treatment.

To control for the effect of body size on neuropil volumes in both species, we included the body size or the CNS volume as a covariate. Our models revealed that, in both species, body size or CNS volume had a significant effect on the volume of the different brain structures except for the SecVN1 neuropil in *P. tepidariorum* (Tables S1 and S2).

### 3.3 Sources of Variation in Neuropil Volume in *M. muscosa* and *P. tepidariorum*

**FIGURE 4:**
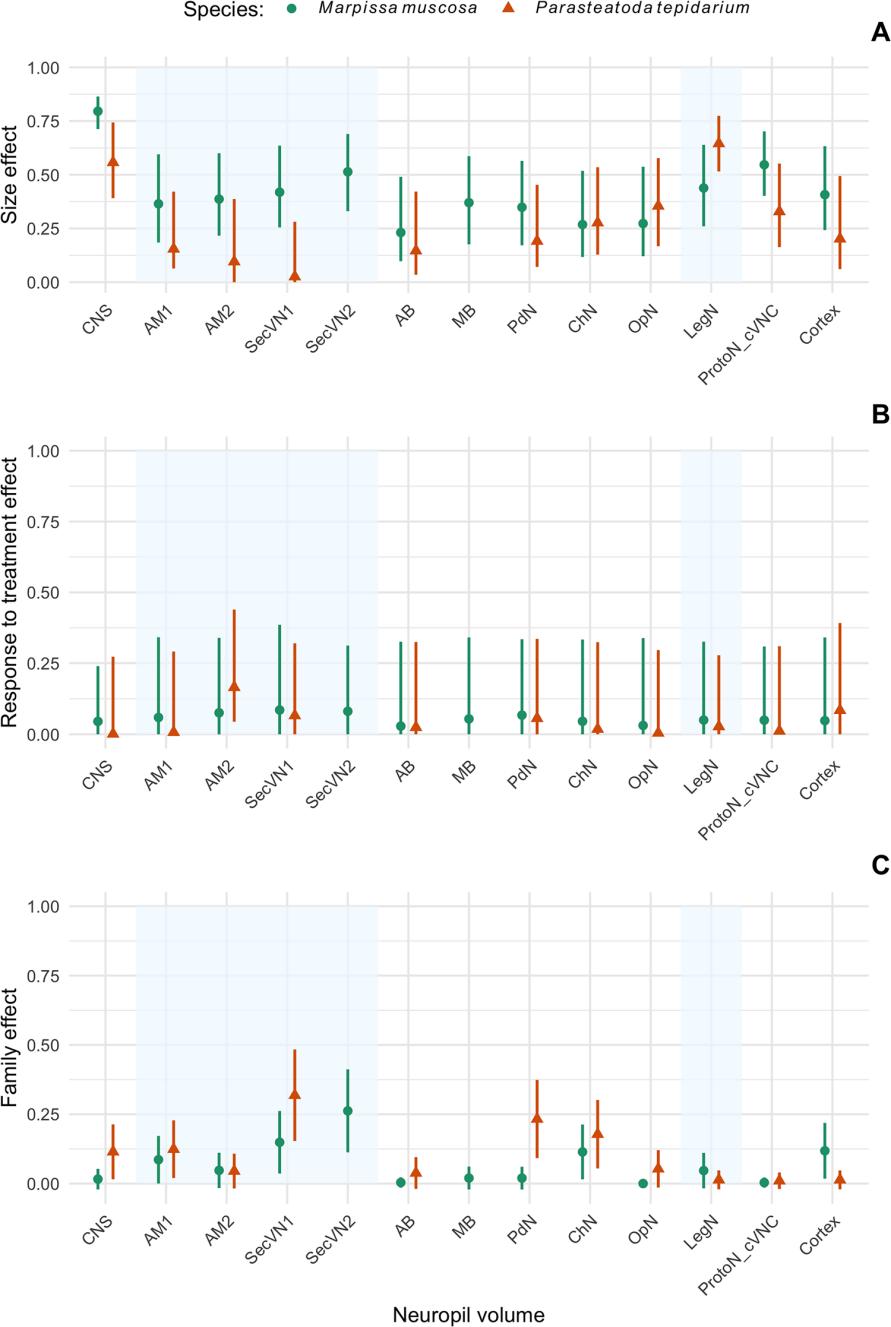
Comparison of the variance compartments for each neuropil volume in each species. The proportion (± 95% CI) of variance explained by (A) the size effect, (B) the treatment effect, and (C) the family effect for each neuropil volume in both *Marpissa muscosa* and *Parasteatoda tepidariorum*. The shaded areas highlight the vision‐related neuropils (AM1, AM2, SecVN1, SecVN2 in *M. muscosa* and AM1, AM2, SecVN1 in *P. tepidariorum*) and the vibration‐related neuropil (LegN). For an explanation of the abbreviations, see Fig. 1.

From our models, we estimated the relative components of variance in neuropil volumes, corresponding to the proportion of variance explained by the CNS size (or body size), the proportion explained by the sensory‐enriched treatments, and the proportion explained by a family effect (genetic and/or maternal effects found in siblings). The phenotypic variance of neuropil volumes is predominantly determined by size, rather than by treatment or the transmitted maternal components. Indeed, the proportion of variance explained by the size of the CNS (or prosoma size for the CNS volume) is large in both species. Conversely, the proportion of variance explained by the treatment or the family component is rather small in both species (Figure 4 and Tables S1 and S2). Nevertheless, the visual neuropils show a larger proportion of variance explained by the family effect compared to nonvisual neuropils (e.g., SecVN1 or SecVN2 vs. nonvisual neuropils, Figure 4C). Furthermore, the family component is larger for the SecVN1 (*M. muscosa* and *P. tepidariorum*) and the SecVN2 (*M. muscosa*) neuropils compared to the AM2 neuropil in both species. The family effect also shows a larger proportion of variance for the PdN and ChN neuropils compared to nonvisual neuropils (e.g., AB, Cortex, ProtoN_VNC) or compared to the LegN neuropil. Additionally, we found an interspecific difference for the PdN neuropil, exhibiting a larger family component in *P. tepidariorum* than in *M. muscosa*.

## 4 Discussion

We reared spiders of a cursorial and a web‐building spider species under different environmental conditions that provided visual or vibratory input or both, and compared the spiders’ brain structures to a deprived control. Our results show moderate effects of sensory enrichment on the neuroanatomy of spiders. Overall, we find that visual neuropils are more sensitive to environmental sensory input than nonvisual neuropils. We analyzed the various sources of variation affecting neuropil volume. Our findings indicate that the sensory enrichment treatments account for little of the variation in neuropil volume. However, we identified a family component that appears to influence the variation of certain neuropil volumes.

### 4.1 Effect of Sensory Enrichment on the Neuropil Volume in *M. muscosa* and *P. tepidariorum*

While we find considerable variation in the volume of all neuropils, the sensory treatments did not induce major volume changes. Although certain neuropils exhibited statistically significant size differences in the treatment groups compared to the control, these effects are challenging to interpret. Notably, the second‐order visual neuropils of the secondary eyes in *M. muscosa* and the second‐order visual neuropils of the principal eyes in *P. tepidariorum* were affected by the VISVIB (in *M. muscosa*) or the VISVIB and VIB (in *P. tepidariorum*) treatment. It is noteworthy that while in *M. muscosa* the visual neuropils of the secondary eyes responded with a volume increase to a mixture of visual and vibratory stimuli, the secondary neuropils of the principal eyes in *P. tepidariorum* showed a decrease in volume. This observation suggests that distinctly different mechanisms may drive the plastic responses of the neuropils in both species.

In the cursorial hunter *M. muscosa*, the visually enhanced environment affected the PdN, whereas environments with vibratory stimuli and the combination of vibratory and visual stimuli did not exert such an effect. This observation seems counterintuitive given that the pedipalps do not receive visual inputs. The visually enhanced treatment may be perceived by the jumping spider as an environment lacking vibratory stimuli, in contrast to a natural, more sensory‐rich environment. This absence of expected vibratory stimuli may cause the individual to increase pedipalp activity. In the stationary hunter *P. tepidariorum*, exposure to vibratory stimulus negatively affected the neuropil volume of the principal eye, while a visually enhanced environment had no effect. The environment that combines visual and vibratory stimuli also affected the size of the secondary eye neuropil. This pattern is similarly counterintuitive and suggests that in *P. tepidariorum*, the perception of mechanosensory stimuli may also activate visual regions, indicating that sensory perception may be more multimodal than previously assumed.

The impact of environmental conditions is overall very small in the present study, which contrasts with the findings of a previous investigation on *M. muscosa*. Steinhoff et al. (2018) found differences in the volume of brain structures (protocerebral neuropil, arcuate, and MB) when the spiders were subjected to different environmental enrichment treatments (physically or socially enriched). Possibly, the sensory enrichment effects in our study were small and/or obscured by the high variability in the volumes of the brain structures. Interestingly, the brain volumes of spiders reared in the laboratory under constant and predictable feeding regimes showed a much broader within‐group variability than that of wild‐caught *M. muscosa* (Steinhoff et al. 2018). This higher variability may result from the regular feeding regime in the laboratory that allows relaxed allocation trade‐offs. Indeed, differences in rearing conditions and feeding regimes are known to shape brain structure volumes (Buchanan et al. 2013; Mery and Burns 2010).

The weak effect of the environmental treatments observed in this study may also be attributed to the characteristics of the stimuli to which the spiders were exposed. First, the enrichment treatments considered may constitute sensorily impoverished and constrained environments for a spider compared to a natural environment. As a consequence, the various treatments may represent a spectrum of deprived environments that failed to elicit distinct changes in brain development. Second, the treatments may not adequately represent natural stimulation. For example, the vibratory stimulus applied involved a vibration of the entire box. Although this may induce vibrations in the web, it may not accurately simulate the vibrations produced by prey entangled in the web. Consequently, these vibrations may have been perceived by the spider as disturbance and may not have elicited a predatory response. Apart from these considerations, changes in the wiring of neuropils are conceivable, which may not have a measurable impact on their volumes (Chittka and Niven 2009) and cannot be detected using the methods employed in our study.

### 4.2 Sources of Variation in Neuropil Volume in *M. muscosa* and *P. tepidariorum*

Our analysis of the relative sources of variation in volume of the different brain structures indicates that, among the three variance components considered (treatment, size, family), the size of the individual is the most significant determinant, while the sensory‐enriched treatments account for a small proportion of variance. The family effect emerges as an interesting source of variation in neuropil volumes. Notably, this study finds that the family effect shows patterns associated with sensory neuropils and that it also affects variation in neuropils that are not directly linked to vision or vibration (e.g., CNS, AB, MB, Cortex, ProtoN_VNC). However, this family effect represents a larger proportion of variance for the vision‐related neuropils in both species (especially SecVN1 and SecVN2). Also, in *P. tepidariorum*, the cheliceral (ChN) and the PdNs show a larger family effect compared to nonsensory‐related neuropils or to the LegN. Thus, taken together, the volume of sensory‐related neuropils may be affected by the family effect to a larger extent compared to other neuropils. Although we cannot specify what constitutes the family effect in our study, it relates to a greater similarity of individuals from the same mother. This similarity may result from additive genetic variation, maternal effects, or from the shared environment in the egg sac. A genetic basis of sensory‐related neuropil volume would suggest that selection could shape their absolute and relative dimensions, as well as their plasticity patterns in response to environmental conditions (Axelrod et al. 2025; Croston et al. 2015). A nongenetic basis of the family effect suggests that environmental conditions early in life may shape sensory‐related neuropil volume. For example, maternal investment in the egg may represent a constraint on brain development (Martin 1996; Van Schaik et al. 2023). Alternatively, an effect of the early environment, such as the shared environment of the egg sac, suggests a plasticity window occurring in the early stages of brain development rather than at adulthood. This may explain why we found that sensory‐related neuropil volume is determined by the family effect to a larger extent than by sensory enrichment or deprivation.

## 5 Conclusion

In our investigation, we subjected two spider species with distinct lifestyles (cursorial vs. stationary hunters) to various sensory environments during their developmental stages. Neither visual, vibratory, nor a combination of both stimuli elicited a discernible pattern in the volume of their neuropils, demonstrating that direct causal relationships are challenging to establish. However, upon estimating the relative significance of the different sources of variation that may influence neuropil volume, we observed a pattern that exhibited similar changes in neuropil volume among siblings, particularly in neuropils that process primary sensory information. Consequently, a genetic, maternal, or sibling effect appears plausible and warrants further investigation.

## Author Contributions

Gabriele Uhl and Philip O. M. Steinhoff conceived and designed the study, Philip O. M. Steinhoff performed the experiments, Pierick Mouginot and Philip O. M. Steinhoff analyzed the results. Philip O. M. Steinhoff wrote the first draft of the manuscript. All authors contributed to writing and editing. Gabriele Uhl supervised the project and secured funding.

## Funding

This study was supported by Deutsche Forschungsgemeinschaft (Grant/Award Numbers: INST 292/119‐1 FUGG, INST 292/120‐1 FUGG, UH87_419146971).

## Supporting information




**Figure S1** Examples of visual stimuli used for video playback.
**Figure S2** Examples of vibratory stimuli used for vibration playback.
**Figure S3** Examples of the output images from the MicroCT scans.
**Table S1** Model estimates of neuropil volumes in *Marpissa muscosa*.
**Table S2** Model estimates of neuropil volumes in *Parasteatoda tepidariorum*.

## Data Availability

The data that support the findings of this study are openly available from the University of Greifswald at ckan.fdm.uni‐greifswald.de/dataset/sensory‐enrichment‐deprivation‐spiders
