# Supplementary material for: Sensory Enrichment and Deprivation During Development: Limited Effects on the Volumes of CNS Neuropils in Two Spiders With Different Ecology
Source: J Comp Neurol. 2025 Nov 20;533(11):e70102. doi: 10.1002/cne.70102 (PMC12635599; doi:10.1002/cne.70102)
Supplement: Supplementary file 1 — Figure S1 Examples of visual stimuli used for video playback. Figure S2 Examples of vibratory stimuli used for vibration playback. Figure S3 Examples of the output images from the MicroCT scans. Table S1 Model estimates of neuropil volumes in Marpissa muscosa. Table S2 Model estimates of neuropil volumes in Parasteatoda tepidariorum. [file CNE-533-e70102-s001.docx]

**Supplementary Materials**


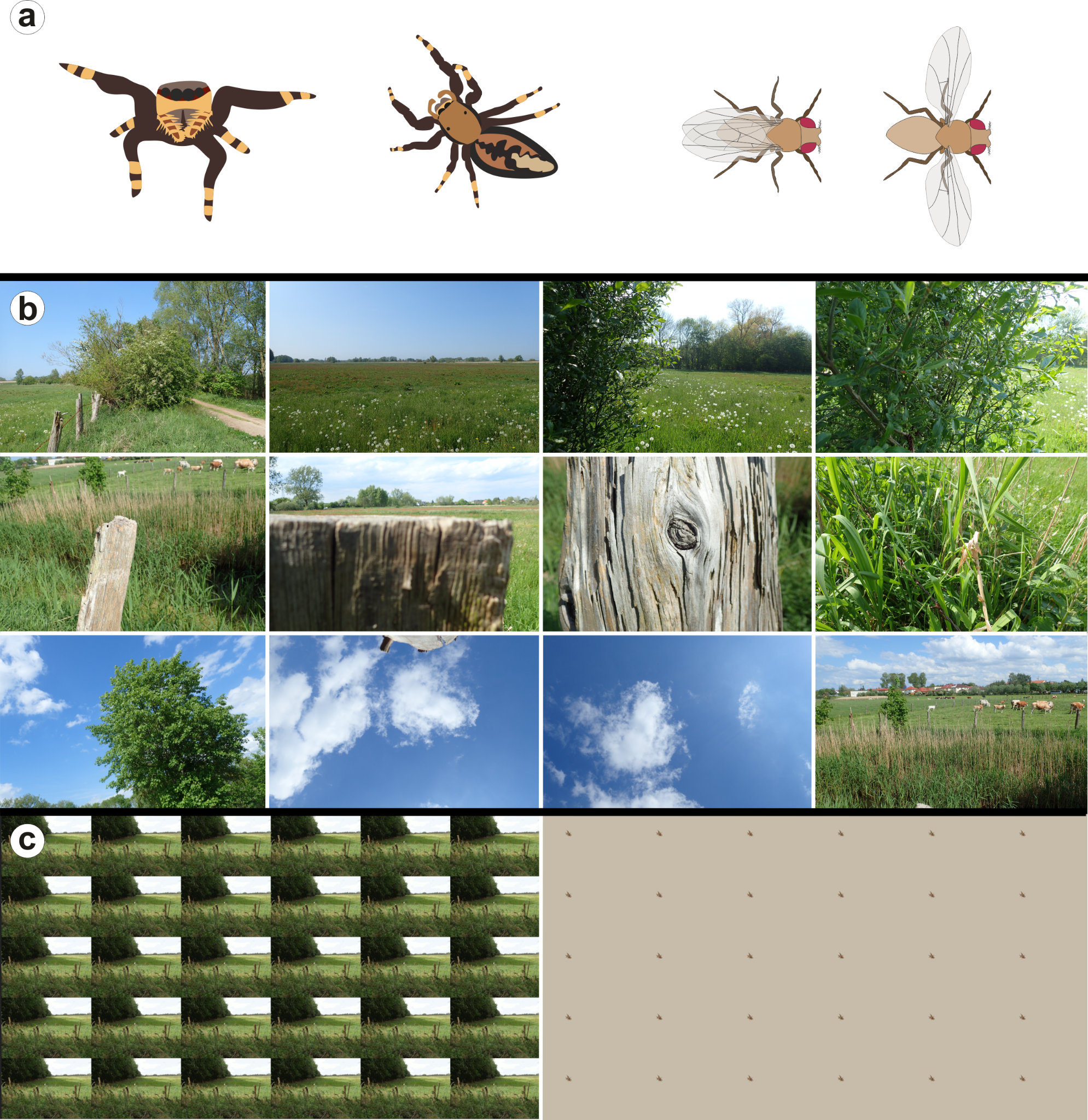


**Figure S1** Examples of visual stimuli used for video playback. (a) Drawings of jumping spiders and flies were prepared according to Menda et al. (2014) and used as individual frames in videos. (b) Examples of environmental images used for playback of still images with either 10 sec. or 1 min. intervals. (c) Examples of mosaic arrangement of videos as played to 30 individual spiders synchronously on 17’’ monitors.


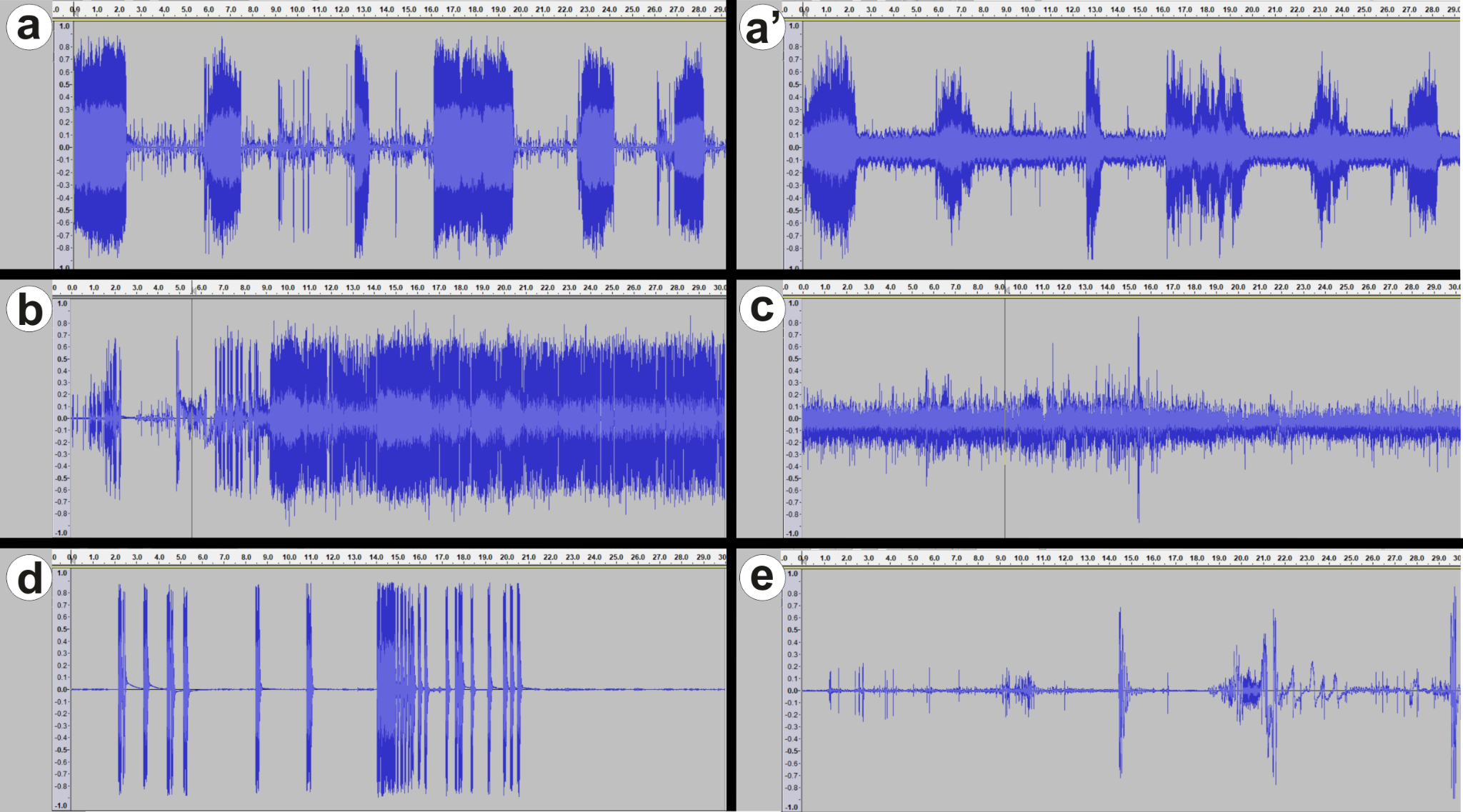


**Figure S2** Examples of vibratory stimuli used for vibration playback, waveform visualization with x axis showing the time in seconds and y axis the amplitude (0=silent, -1,+1 maximum noise). For better visibility, only the first 30 seconds of each recording are shown. (a) Waveform showing “*Calliphora* buzz” as recorded directly from the fly. (a’) “*Calliphora buzz”* control recorded from the base of the spider rearing box when played via a piezo-ceramic element shows similar pattern and amplitude to original recording. (b) Waveform showing *“Lucilia buzz”* vibration. (c) Waveform showing *“Drosophila walking”* vibration. (d) Waveform showing *“Noise with regular amplitude”* vibration. (e) Waveform showing *“Noise with irregular amplitude”* vibration.


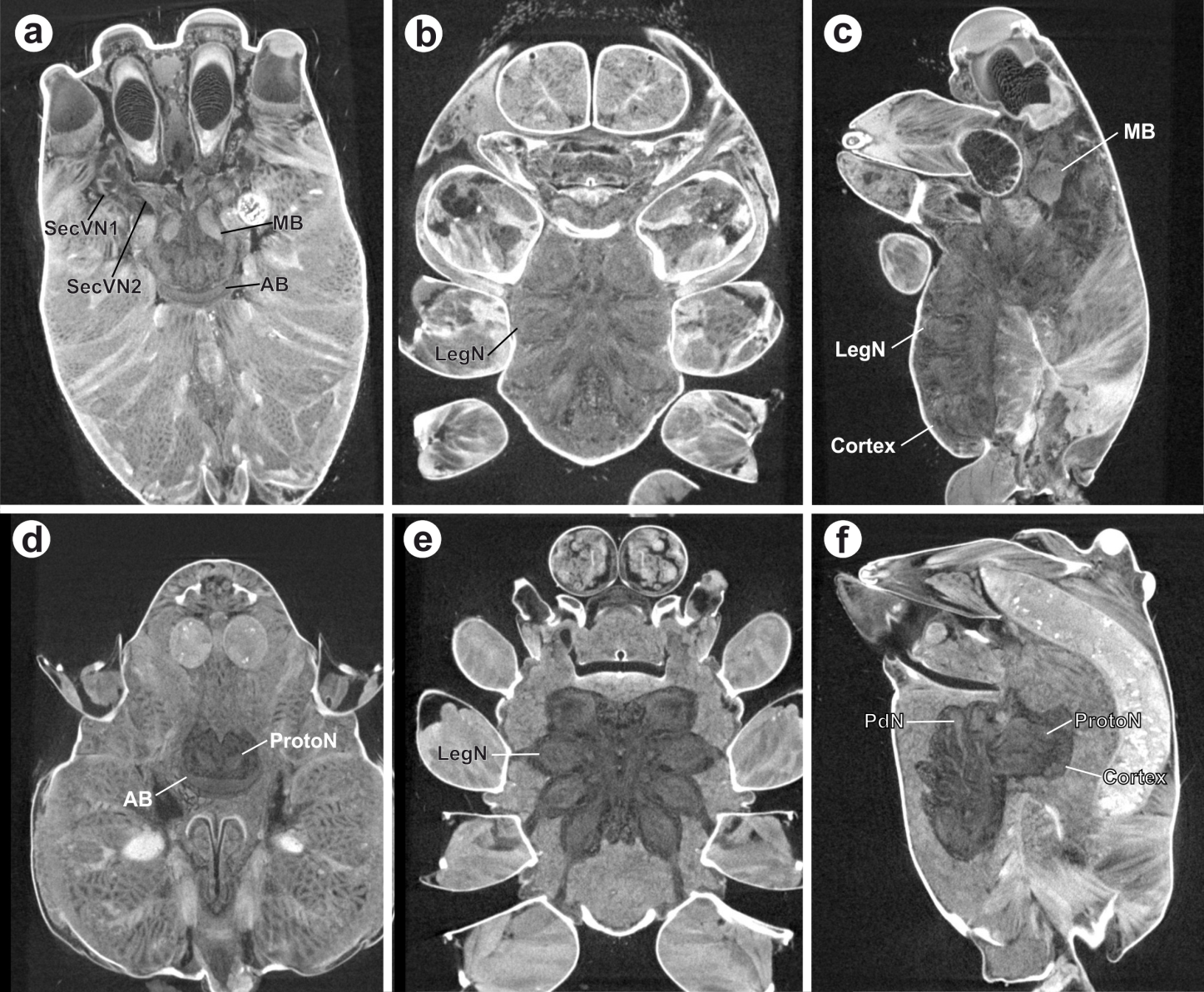


**Figure S3** Examples of the output images from the MicroCT scans of *M. muscosa* (a,b,c) and *P. tepidariorum* (c,d,f) from which volume reconstruction were performed.

Abbreviations: AB, arcuate body; LegN, leg neuropils; MB, mushroom bodies; PdN, pedipalpal neuropil; SecVis1, first-order visual neuropils of all secondary eyes; SecVis2, second-order visual neuropils of all secondary eyes; ProtoN, protocerebral neuropil.

**Table S1. Model estimates of neuropil volumes in *Marpissa* *muscosa*.**

Model estimates of the treatments (vibration, visual, visual and vibration compared to the control) with the 95% confidence interval, Observer effect (with SE and p-value), slope estimate (with SE and p-value), polynomials of degree 2 and 3 (with their SE and p-values). Part of variances explained by the models: marginal part corresponding to the variance explained by the fixed effects only (marg.R.sq with 95% CI), part of variance explained by the random effect (rand.part.var with 95% CI), part of variance explained by the body size or CNS volume (including polynomials, with their 95% CI) and part of variance explained by the treatment (with 95% CI).

| **response** | **vib** | **CIl vib** | **CIu vib** | **vis** | **CIl vis** | **CIu vis** | **visvib** | **CIlvisvib** | **CIuvisvib** | **Observer** | **S.E.Obs** | **p.val.Obs** |
| --- | --- | --- | --- | --- | --- | --- | --- | --- | --- | --- | --- | --- |
| **CNS** | -0.204 | -0.450 | 0.043 | -0.105 | -0.355 | 0.146 | 0.180 | -0.062 | 0.421 | 1.551 | 0.259 | 0.000 |
| **AM1** | -0.285 | -0.952 | 0.382 | 0.231 | -0.476 | 0.938 | 0.219 | -0.481 | 0.919 | 0.220 | 0.349 | 0.532 |
| **AM2** | 0.047 | -0.590 | 0.684 | 0.538 | -0.139 | 1.214 | 0.553 | -0.116 | 1.222 | 0.204 | 0.350 | 0.563 |
| **SecVis1** | 0.163 | -0.428 | 0.754 | 0.524 | -0.102 | 1.150 | 0.690 | 0.071 | 1.308 | 0.465 | 0.344 | 0.183 |
| **SecVis2** | 0.380 | -0.162 | 0.921 | 0.456 | -0.121 | 1.033 | 0.617 | 0.051 | 1.183 | 0.311 | 0.348 | 0.377 |
| **AB** | 0.253 | -0.421 | 0.926 | 0.424 | -0.270 | 1.119 | 0.358 | -0.330 | 1.045 | -0.468 | 0.309 | 0.137 |
| **MB** | 0.453 | -0.176 | 1.082 | 0.577 | -0.093 | 1.247 | 0.594 | -0.068 | 1.256 | 0.232 | 0.349 | 0.510 |
| **PdN** | 0.333 | -0.297 | 0.964 | 0.731 | 0.059 | 1.402 | 0.411 | -0.252 | 1.074 | 0.191 | 0.350 | 0.587 |
| **ChN** | -0.362 | -1.048 | 0.323 | 0.188 | -0.538 | 0.915 | 0.124 | -0.595 | 0.844 | 0.511 | 0.342 | 0.143 |
| **LegN** | 0.299 | -0.228 | 0.826 | 0.369 | -0.212 | 0.950 | 0.453 | -0.103 | 1.009 | 0.415 | 0.306 | 0.181 |
| **OpN** | 0.423 | -0.227 | 1.073 | 0.073 | -0.624 | 0.770 | 0.144 | -0.546 | 0.833 | 0.539 | 0.312 | 0.091 |
| **ProtoN VNC** | -0.079 | -0.345 | 0.186 | 0.215 | -0.070 | 0.501 | 0.168 | -0.118 | 0.455 | -1.295 | 0.186 | 0.000 |
| **Cortex** | 0.394 | -0.233 | 1.021 | 0.175 | -0.489 | 0.839 | 0.523 | -0.134 | 1.180 | 0.277 | 0.348 | 0.431 |

| **response** | **slope** | **S.E.slope** | **p.val.slope** | **poly2** | **S.E.poly2** | **p.val.poly2** | **poly3** | **S.E.poly3** | **p.val.poly3** |
| --- | --- | --- | --- | --- | --- | --- | --- | --- | --- |
| **CNS** | 4.691 | 0.325 | 0.000 | -0.675 | 0.285 | 0.024 | NA | NA | NA |
| **AM1** | 4.391 | 0.890 | 0.000 | 0.469 | 0.863 | 0.590 | NA | NA | NA |
| **AM2** | 4.478 | 0.832 | 0.000 | -0.121 | 0.822 | 0.883 | NA | NA | NA |
| **SecVis1** | 4.466 | 0.819 | 0.000 | 1.323 | 0.768 | 0.094 | NA | NA | NA |
| **SecVis2** | 5.579 | 0.776 | 0.000 | -0.201 | 0.705 | 0.778 | 0.810 | 0.636 | 0.213 |
| **AB** | 3.010 | 0.822 | 0.001 | -0.363 | 0.813 | 0.657 | NA | NA | NA |
| **MB** | 4.256 | 0.801 | 0.000 | -0.713 | 0.810 | 0.385 | NA | NA | NA |
| **PdN** | 4.176 | 0.803 | 0.000 | -0.488 | 0.812 | 0.551 | NA | NA | NA |
| **ChN** | 3.627 | 0.925 | 0.000 | -0.977 | 0.888 | 0.279 | NA | NA | NA |
| **LegN** | 3.961 | 0.705 | 0.000 | -0.838 | 0.693 | 0.234 | NA | NA | NA |
| **OpN** | 3.058 | 0.779 | 0.001 | -1.266 | 0.818 | 0.130 | -0.113 | 0.766 | 0.884 |
| **ProtoN VNC** | 1.766 | 0.332 | 0.000 | -1.754 | 0.336 | 0.000 | 0.870 | 0.316 | 0.009 |
| **Cortex** | 4.659 | 0.854 | 0.000 | -0.372 | 0.813 | 0.650 | NA | NA | NA |

| **response** | **marg.R.sq** | **CIl.mar** | **CIu.mar** | **rand.part.var** | **CIl.rand** | **CIu.rand** | **slope** | **CIl.slope** | **CIu.slope** | **treat** | **CIl.treat** | **CIu.treat** |
| --- | --- | --- | --- | --- | --- | --- | --- | --- | --- | --- | --- | --- |
| **CNS** | 0.833 | 0.750 | 0.903 | 0.016 | -0.021 | 0.053 | 0.796 | 0.713 | 0.864 | 0.045 | 0 | 0.240 |
| **AM1** | 0.394 | 0.215 | 0.620 | 0.086 | 0.000 | 0.172 | 0.365 | 0.185 | 0.595 | 0.059 | 0 | 0.341 |
| **AM2** | 0.447 | 0.284 | 0.650 | 0.048 | -0.016 | 0.111 | 0.387 | 0.216 | 0.600 | 0.076 | 0 | 0.339 |
| **SecVis1** | 0.446 | 0.283 | 0.662 | 0.149 | 0.036 | 0.262 | 0.419 | 0.255 | 0.636 | 0.086 | 0 | 0.386 |
| **SecVis2** | 0.545 | 0.361 | 0.722 | 0.262 | 0.113 | 0.412 | 0.514 | 0.331 | 0.690 | 0.081 | 0 | 0.312 |
| **AB** | 0.281 | 0.154 | 0.534 | 0.004 | -0.015 | 0.023 | 0.231 | 0.098 | 0.490 | 0.029 | 0 | 0.326 |
| **MB** | 0.460 | 0.283 | 0.663 | 0.020 | -0.021 | 0.061 | 0.370 | 0.176 | 0.587 | 0.054 | 0 | 0.341 |
| **PdN** | 0.463 | 0.301 | 0.670 | 0.020 | -0.021 | 0.061 | 0.349 | 0.172 | 0.564 | 0.068 | 0 | 0.335 |
| **ChN** | 0.321 | 0.174 | 0.563 | 0.114 | 0.015 | 0.213 | 0.268 | 0.117 | 0.518 | 0.046 | 0 | 0.333 |
| **LegN** | 0.490 | 0.317 | 0.687 | 0.047 | -0.017 | 0.111 | 0.438 | 0.260 | 0.639 | 0.050 | 0 | 0.326 |
| **OpN** | 0.345 | 0.200 | 0.597 | 0.000 | -0.004 | 0.004 | 0.273 | 0.121 | 0.537 | 0.031 | 0 | 0.338 |
| **ProtoN VNC** | 0.666 | 0.530 | 0.805 | 0.004 | -0.015 | 0.022 | 0.547 | 0.402 | 0.702 | 0.050 | 0 | 0.309 |
| **Cortex** | 0.444 | 0.278 | 0.664 | 0.118 | 0.018 | 0.219 | 0.407 | 0.243 | 0.633 | 0.048 | 0 | 0.341 |

**Table S2. Model estimates of neuropil volumes in *Parasteatoda tepidariorum***

Model estimates of the treatments (vibration, visual, visual and vibration compared to the control) with the 95% confidence interval, Observer effect (with SE and p-value), slope estimate (with SE and p-value), polynomials of degree 2 and 3 (with their SE and p-values). Part of variances explained by the models: marginal part corresponding to the variance explained by the fixed effects only (marg.R.sq with 95% CI), part of variance explained by the random effect (rand.part.var with 95% CI), part of variance explained by the body size or CNS volume (including polynomials, with their 95% CI) and part of variance explained by the treatment (with 95% CI).

| **response** | **vib** | **CIl vib** | **CIu vib** | **vis** | **CIl vis** | **CIu vis** | **visvib** | **CIlvisvib** | **CIuvisvib** | **Observer** | **S.E.Obs** | **p.val.Obs** |
| --- | --- | --- | --- | --- | --- | --- | --- | --- | --- | --- | --- | --- |
| **CNS** | 0.246 | -0.271 | 0.763 | 0.169 | -0.299 | 0.637 | 0.036 | -0.438 | 0.510 | -0.230 | 0.324 | 0.482 |
| **Vis1** | 0.143 | -0.610 | 0.895 | 0.204 | -0.495 | 0.903 | 0.180 | -0.515 | 0.875 | -0.374 | 0.298 | 0.216 |
| **Vis2** | 0.089 | -0.646 | 0.824 | 0.423 | -0.240 | 1.085 | 0.736 | 0.066 | 1.407 | -0.297 | 0.323 | 0.362 |
| **Vis3** | -1.164 | -1.912 | -0.416 | -0.382 | -1.092 | 0.328 | -0.747 | -1.446 | -0.047 | -0.017 | 0.311 | 0.957 |
| **AB** | -0.208 | -0.933 | 0.516 | 0.177 | -0.489 | 0.842 | 0.008 | -0.668 | 0.684 | -1.044 | 0.284 | 0.001 |
| **PdN** | 0.098 | -0.642 | 0.838 | 0.132 | -0.538 | 0.802 | -0.508 | -1.186 | 0.171 | -0.152 | 0.325 | 0.643 |
| **ChN** | 0.054 | -0.674 | 0.783 | 0.310 | -0.352 | 0.971 | 0.021 | -0.649 | 0.692 | 0.202 | 0.324 | 0.538 |
| **LegN** | 0.110 | -0.414 | 0.634 | 0.035 | -0.447 | 0.517 | -0.147 | -0.637 | 0.342 | -0.232 | 0.324 | 0.478 |
| **OpN** | 0.120 | -0.552 | 0.792 | 0.085 | -0.531 | 0.700 | 0.224 | -0.401 | 0.849 | 0.661 | 0.310 | 0.039 |
| **ProtoN VNC** | 0.03 | -0.553 | 0.623 | -0.073 | -0.618 | 0.472 | -0.066 | -0.618 | 0.486 | 0.439 | 0.272 | 0.113 |
| **Cortex** | 0.068 | -0.734 | 0.871 | 0.257 | -0.482 | 0.996 | -0.593 | -1.345 | 0.158 | 0.136 | 0.325 | 0.677 |

| **response** | **slope** | **S.E.slope** | **p.val.slope** | **poly2** | **S.E.poly2** | **p.val.poly2** | **poly3** | **S.E.poly3** |
| --- | --- | --- | --- | --- | --- | --- | --- | --- |
| **CNS** | 4.930 | 0.655 | 0.000 | -0.649 | 0.692 | 0.354 | NA | NA |
| **Vis1** | 2.511 | 0.914 | 0.009 | -0.362 | 0.999 | 0.719 | NA | NA |
| **Vis2** | 1.079 | 0.947 | 0.262 | -0.390 | 0.995 | 0.697 | NA | NA |
| **Vis3** | 1.820 | 0.871 | 0.045 | 1.180 | 0.960 | 0.227 | -0.271 | 0.876 |
| **AB** | 2.002 | 0.847 | 0.024 | -1.281 | 0.927 | 0.175 | NA | NA |
| **PdN** | 2.786 | 0.936 | 0.005 | -1.462 | 0.990 | 0.148 | NA | NA |
| **ChN** | 3.703 | 0.910 | 0.000 | 0.164 | 0.967 | 0.867 | NA | NA |
| **LegN** | 5.146 | 0.607 | 0.000 | -1.921 | 0.667 | 0.006 | NA | NA |
| **OpN** | 3.190 | 0.798 | 0.000 | -2.610 | 0.867 | 0.005 | NA | NA |
| **ProtoN VNC** | 3.216 | 0.685 | 0.000 | -0.924 | 0.724 | 0.212 | NA | NA |
| **Cortex** | 2.568 | 0.917 | 0.008 | -2.047 | 1.015 | 0.051 | NA | NA |

| **response** | **marg.R.sq** | **CIl.mar** | **CIu.mar** | **rand.part.var** | **CIl.rand** | **CIu.rand** | **slope** | **CIl.slope** | **CIu.slope** | **treat** | **CIl.treat** | **CIu.treat** |
| --- | --- | --- | --- | --- | --- | --- | --- | --- | --- | --- | --- | --- |
| **CNS** | 0.569 | 0.401 | 0.740 | 0.115 | 0.016 | 0.214 | 0.558 | 0.392 | 0.731 | 0.001 | 0.000 | 0.264 |
| **Vis1** | 0.160 | 0.065 | 0.446 | 0.124 | 0.020 | 0.228 | 0.155 | 0.059 | 0.442 | 0.007 | 0.000 | 0.316 |
| **Vis2** | 0.115 | 0.048 | 0.387 | 0.319 | 0.154 | 0.484 | 0.026 | 0.000 | 0.295 | 0.066 | 0.000 | 0.334 |
| **Vis3** | 0.258 | 0.143 | 0.536 | 0.045 | -0.018 | 0.108 | 0.096 | 0.000 | 0.395 | 0.166 | 0.041 | 0.456 |
| **AB** | 0.189 | 0.078 | 0.444 | 0.038 | -0.019 | 0.095 | 0.147 | 0.035 | 0.406 | 0.025 | 0.000 | 0.305 |
| **PdN** | 0.223 | 0.095 | 0.477 | 0.233 | 0.092 | 0.374 | 0.191 | 0.061 | 0.451 | 0.056 | 0.000 | 0.337 |
| **ChN** | 0.279 | 0.138 | 0.528 | 0.178 | 0.055 | 0.301 | 0.277 | 0.136 | 0.527 | 0.018 | 0.000 | 0.319 |
| **LegN** | 0.656 | 0.525 | 0.787 | 0.014 | -0.020 | 0.048 | 0.645 | 0.514 | 0.776 | 0.027 | 0.000 | 0.284 |
| **OpN** | 0.399 | 0.241 | 0.616 | 0.053 | -0.014 | 0.121 | 0.355 | 0.190 | 0.580 | 0.005 | 0.000 | 0.296 |
| **ProtoN VNC** | 0.381 | 0.220 | 0.605 | 0.010 | -0.020 | 0.040 | 0.329 | 0.163 | 0.552 | 0.012 | 0.000 | 0.310 |
| **Cortex** | 0.260 | 0.129 | 0.509 | 0.014 | -0.020 | 0.047 | 0.202 | 0.060 | 0.459 | 0.085 | 0.000 | 0.360 |
